# Maternal Body Mass Index and Risk of Autism Spectrum Disorders in Offspring: A Meta-analysis

**DOI:** 10.1038/srep34248

**Published:** 2016-09-30

**Authors:** Ying Wang, Shiming Tang, Shunsheng Xu, Shenhong Weng, Zhongchun Liu

**Affiliations:** 1 Mental Health Center, Renmin Hospital of Wuhan University, Jiefang Road 238#, Wuchang District, Wuhan 430060, P. R. China; 2 Hubei Provincial Mental Health Center, Jiefang Road 238#, Wuchang District, Wuhan 430060, P. R. China; 3 Department of Psychiatry, Institution of Neuropsychiatry Research, Renmin Hospital of Wuhan University, Jiefang Road 238#, Wuchang District, Wuhan 430060, P. R. China

## Abstract

Controversial results of the association between maternal body mass index (BMI) and risk of autism spectrum disorder (ASD) in offspring were reported among several studies. This meta-analysis was conducted to estimate the overall association between maternal BMI and risk of ASD in offspring. PubMed, EMBASE, Web of Science, and the Cochrane Library were searched until January 2016. Cohort and case-control studies addressing the association between maternal BMI and risk of ASD in offspring were included. We used random-effect models to estimate the summary relative risks (RRs), we also performed a dose-response meta-analysis to estimate the trend from the correlated log RR estimates across levels of BMI quantitatively. Totally, 6 cohort studies and 1 case-control study involving 8,403 cases and 509,167 participants were included for analysis. The summary RR (95% confidence interval) for ASD in offspring in relation to maternal underweight, overweight, and obesity vs. normal weight during pre-pregnancy or pregnancy, was 1.07 (0.93, 1.23), 1.28 (1.19, 1.36) and 1.36 (1.03, 1.78), respectively. A linear dose-response relationship was found, with a pooled RR of 1.16 (1.01, 1.33) for each 5 kg/m^2^. increment in maternal BMI. The present study suggests that excessive maternal BMI is associated with increased ASD risk in offspring.

Autism spectrum disorders (ASD) are a group of complex neurodevelopmental disorders characterized by impairments in social interaction and communications, as well as restricted and repetitive behaviors^1^. The reported prevalence of ASD has been increasing since the 1990 s, and it is projected to be 1 person in 132 in 2010 worldwide^2^. While the etiology of ASD still remains unclear, both genetic and environmental factors are thought to play a role^3^. Among diverse factors, maternal conditions during pre-pregnancy or pregnancy are increasingly being recognized as potential risk factors for ASD. Evidence from meta-analysis indicated that maternal advanced age^4^, diabetes^5^, and antidepressants use^6^ during pregnancy was associated with elevated ASD risk in offspring. Maternal obesity, which has become a global health problem, has also been studied by several epidemiological studies to examine a possible association with ASD. However, the results were inconclusive. For example, while the study by Xaing *et al*.^7^ and Garndner *et al*.^8^ found a positive association between maternal obesity and ASD risk, the study by Moss *et al*.^9^ found no statistically significant results.

A recent meta-analysis by Li *et al*.^10^ reviewed the studies regarding maternal obesity and ASD risk and found a positive relationship of maternal obesity with ASD risk. Nevertheless, they included one study using body weight as cut-off point for obesity which might introduce bias because other included studies ascertain obesity based on body mass index (BMI). Moreover, Li *et al*.^10^ only focused on the effect of maternal obesity on ASD in offspring, but failed to fully assess the potential association of different category of BMI including overweight and underweight with ASD risk, and they failed to explore a possible dose-response relation of BMI and ASD risk because of the insufficient data. In this study, we aimed to systematically assess the association between maternal BMI (underweight, overweight and obesity) and ASD risk in offspring, trying to find a dose-response relation between them.

## Results

### Literature search and selection

We retrieved 1525 records using the search strategy. After removal of duplicate literatures, 1123 articles were left for screening. From screening the titles and abstracts, 1111 articles were excluded as they were not clearly relevant. After evaluating the full text of the remaining 12 literatures, we further excluded 5 studies^11^,^12^,^13^,^14^,^15^ that did not report risk estimates of concern. Finally, 7 studies^7^,^8^,^9^,^14^,^16^,^17^,^18^ were included for analysis (See Supplemental Figure 1).

### Study characteristics

Table 1 lists the characteristics of the included studies, which consisted of 6 cohort studies (5 prospective and 1 retrospective) and 1 case-control studies. There were 8,403 cases and 509,167 participants. While 5 studies were conducted in the US, 2 studies were from Europe (Norway and Sweden). The information about BMI was generally collected during pre-pregnancy, and ASDs were ascertained by standard assessment in most of studies. The methodological quality of all studies was good, with a range score of 7–9 (average: 8.1) (See Supplemental Table 1).

### Categorical meta-analysis

**Figure 1:**
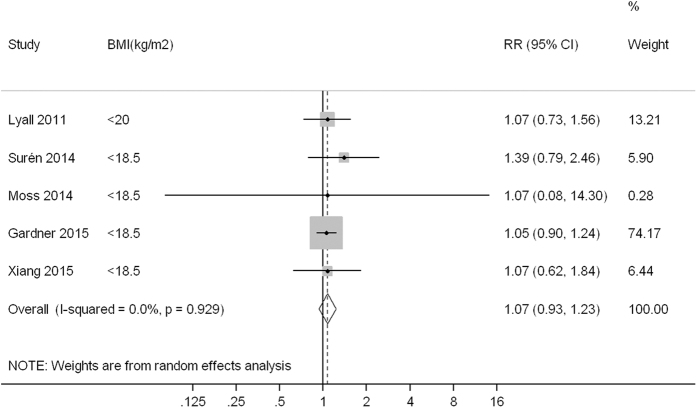
A forest plot of the association between maternal underweight and ASD risk.

**Figure 2:**
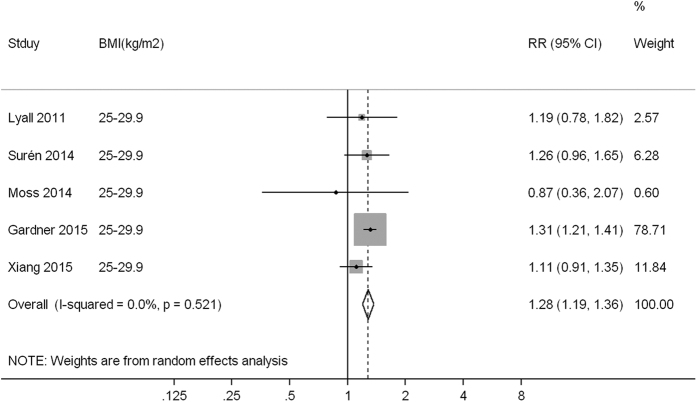
A forest plot of the association between maternal overweight and ASD risk.

**Figure 3:**
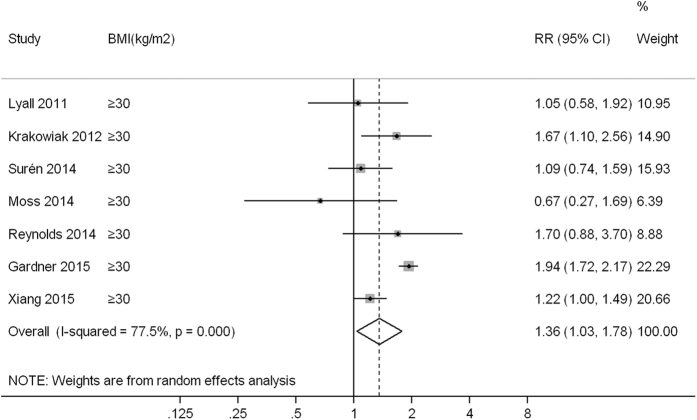
A forest plot of the association between maternal obesity and ASD risk.

Compared to children born to mothers with normal weight (or non-obese weight), the pooled RR (95% confidence interval, CI) of ASD was 1.07 (0.93, 1.23), 1.28 (1.19, 1.36) and 1.36 (1.03, 1.78) for children born to mothers with underweight, overweight and obesity, with low heterogeneity (*I*^*2*^ = 0.0%, *P* = 0.929), low heterogeneity (*I*^*2*^ = 0.0%, *P* = 0.521) and high heterogeneity (*I*^*2*^ = 77.5%, *P* < 0.001) observed across studies, respectively (Figs 1,2 and 3).

### Dose-response meta-analysis

**Figure 4:**
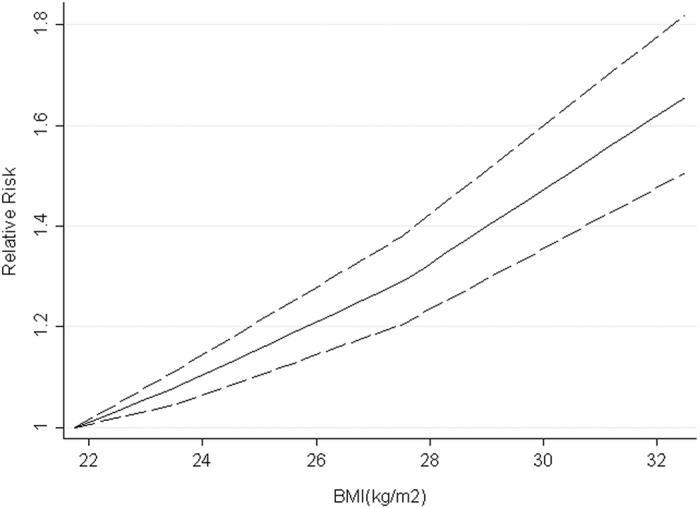
Dose-response analysis for maternal BMI and ASD risk. The solid line and the long dashed lines represent the estimated relative risk and corresponding 95%CI, respectively.

4 studies were included for dose-response analysis. A linear dose-response relationship was found between maternal BMI and risk of ASD (*P*-nonlinearity = 0.673). Compared with children born to mothers in normal weight, the pooled RR for ASD in offspring was 1.16 (1.01, 1.33) for each 5 kg/m^2^. increment in maternal BMI (Fig. 4).

### Subgroup and sensitivity analysis

Table 2 summarizes the main findings of the meta-analysis according to categories of maternal BMI versus normal weight stratified by study design, study location, and adjustment for confounders. For children born to underweight mothers, the summary risk estimates for ASD were generally comparable and similar to that of overall result when stratified by different factors. Stronger associations between maternal overweight/obesity and ASD risk were found in European studies, and in which BMI were measured at first trimester and ASD were ascertained by standard assessment. When stratified by certain confounders, stronger associations were found in studies adjusted for maternal age, child sex, and birth year than studies that did not adjust for such confounders. In sensitivity analysis, the summary RRs did not substantially changed for different categories of maternal BMI before and after elimination of each study in the meta-analyses, which indicated that our results were robust.

### Publication bias

There was no evidence of significant publication bias among the included studies according to Begg’s test and Egger’s test (all *P* > 0.05).

## Discussion

In this meta-analysis of observational studies, we explored the effect of maternal BMI (underweight, overweight or obesity) during pre-pregnancy or pregnancy on ASD risk in offspring. Compared with children whose mothers were at normal weight, children born to overweight and obese mothers have a 28% and 36% higher risk of developing ASD, respectively. Maternal underweight was not associated with increased ASD risk. A linear dose-response relationship was found, with the risk of ASD increasing by 16% for each 5 kg/m^2^ increment in maternal BMI compared with that of normal weight. ASD is one of the most common and severe neurodevelopmental disorders which is lifelong. It not only significantly impacts upon the individuals, but also has long-term implications for their families, as well as for the provision of education and habilitative services^19^. Some behavioral treatments have been suggested to produce positive short-term benefits^19^. Nevertheless, given that the noticeable clinical and genetic heterogeneity between affected individuals, the lack of reliable diagnostic biomarkers, and the unrevealed underlying pathophysiological mechanisms, there are still no effective treatments for the core symptoms of ASD^20^. It is therefore crucial to identify related risk factors and to prevent ASD in the primary step.

Along with the nearly doubled world’s obesity rate between 1980 and 2008, the prevalence of ASD has also been increasing rapidly during the same period. While elevated awareness and updated diagnostic criteria of ASD might contribute to its increased prevalence, it is possible that the obesity epidemic may also play a role, which is supported by the results of our meta-analysis. The present findings provide strong persuasion for women to keep appropriate weight during pre-pregnancy or pregnancy, so as to reduce ASD risk in their offspring.

Although the causal pathway remains to be elucidated, the effects of maternal BMI on ASD may be explained by several hypothesizes. Of the proposed mechanisms, inflammation is the most frequently mentioned one to explain the association of maternal BMI and ASD. It is observed that obese women had higher levels of C-reactive protein in the plasma compared with normal weight pregnant women^21^. In addition, increased CD68 + and CD14 + cells with elevated expression of inflammatory cytokines including tumor necrosis factor-alpha, interleukin-6, and interleukin-1 in the placentas have also been found in obese pregnant mothers^22^,^23^. As placenta inflammation is associated with neonatal brain damage and can induce a systemic fetal inflammatory response which may contribute to white matter injury in the fetal brain^23^, it is plausible that the risk of mental disorders increases for children born to mother with overweight/obesity.

Obesity is a significant risk factor for diabetes, while maternal diabetes itself significantly increase the risk of ASD in the offspring^5^. Hyperglycemia, as a consequence of maternal diabetes, is supposed to increase ASD risk in offspring through several mechanisms, such as hypoxia in the fetus, increased free-radical production and impaired antioxidant defense system^24^,^25^,^26^. Dietary and nutrition factors plays important role in the development of both obesity and diabetes. However, nutritional factors such as fat intake, vitamins may also contribute to the development of ASD^27^,^28^. Nevertheless, the association between maternal obesity and ASD risk in offspring may be simultaneously mediated by multiple factors and the explicit mechanism needs further elucidation.

This meta-analysis was based on observational studies. Therefore, we cannot exclude potential biases due to other factors which may contribute to ASD. For instance, mothers who have lower BMI will probably have more healthy lifestyles, such as more physical exercise, less intake of salt and saturated fat, and thus they are less likely to be affected by hyperglycemia and diabetes than those who have higher BMI. Although all studies controlled for several factors, including maternal age at baseline, race, child’s sex, and birth year etc, the potential influences from residual or unmeasured confounding cannot be ruled out.

Apart from maternal BMI, paternal obesity was also suggested to be associated with risk of autism in offspring. In two included studies, Gardner *et al*.^8^ and Surén *et al*.^18^ respectively reported a 47% (OR 1.47, 95% CI 1.12–1.92) and 73% (OR 1.73, 95% CI 1.07–2.82) increased risk of fathers with obesity having a child diagnosed with autism, compared with the risk of autism in children of nonobese fathers. One reason of this association might be obesity-related alterations to epigenetic information, which modulate gene expression that is essential for proper development after fertilization^29^. Moreover, the two studies also suggested that when adjusted for paternal and other confounding factors, the risk of autism in children with obese mothers was attenuated. Considering that paternal obesity was not adjusted in other five included studies, the positive association between maternal BMI and risk of autism concluded by this meta-analysis might be overestimated.

Two recent studies^8^,^12^ found that the association between maternal BMI and offspring risk of ASD does not hold when ASD cases were compared to their matched, unaffected siblings. Both the sibling study and the study of paternal BMI suggested that maternal BMI might be a proxy marker for inherited background which potentially explains some of the risk attributable to maternal BMI^8^. An evidence is that the deletion or duplication in a region of chromosome 16p11.2 was indicated to influence susceptibility to autism^30^, meanwhile 16p11.2 deletions were associated with obesity and morbid obesity at or near genome-wide levels of significance, compared to lean/normal weight subjects^31^. Therefore, the potential underlying genetic mechanism regarding maternal BMI and risk of ASD in offspring should be further explored in future studies.

Maternal BMI were self-reported without objective ascertainment in 3 included studies^9^,^17^,^18^. Nevertheless, women tend to overestimate their height and underestimate their weight by self-report^32^,^33^. There was possibility that the risk of ASD in offspring was underestimated as the BMI might be underestimated. In addition, six studies^7^,^9^,^14^,^16^,^17^,^18^ measured maternal BMI at pre-pregnancy while one^8^ assessed it at the first antenatal visit, lacking of a continuous documentation. However, the process of pregnancy often accompanies with weight gain^34^. While excessive prenatal weight gain is suggested to be associated with elevated risk of ASD in offspring^12^, the positive association between maternal BMI and ASD risk might partially be attributed to excessive prenatal weight gain. Nevertheless, besides Gardner *et al*.^8^ adjusted for gestational weight gain in one of their sensitivity analysis, none of other included studies adjusted for weight gain during pregnancy. It is therefore necessary for future studies to exclude the possible effect of prenatal weight gain on ASD risk.

Our meta-analysis included 2 retrospective studies^7^,^16^. Retrospective design may incur several biases, including selection bias and recall bias. The improper selection of cases and controls, the biased collection of exposure and related confounding factors, can both lead to a biased result. Nevertheless, when we restricted study design to prospective cohort studies, the results did not markedly alter, suggesting that study design did not significantly influence the general results.

In conclusion, the present study suggests that excessive maternal BMI is associated with an increased ASD risk in offspring. Approximately, every 5 kg/m^2^ increment in BMI is associated with 16% increased ASD risk. Considering the limited small number of included studies, further well-designed studies are needed to confirm the our findings.

## Methods

### Data sources and searches

We conducted a literature search of PubMed, Embase, Web of Science, and Cochrane Library up to 29 January 2016 for case-control and cohort studies examining the association between maternal BMI and risk of ASD in offspring. The search terms for PubMed were: (body mass index OR overweight OR obesity) AND autism. Similar search strategies were used in other databases. In addition, we screened references list from relevant original papers and review articles to identify further pertinent studies. We applied no language restriction in the process of literature search and selection. Two researchers independently conducted the literature search and disagreement was solved by discussion. The Meta-Analysis of Observational Studies in Epidemiology guidelines^35^ was followed to conduct this meta-analysis.

### Study selection

Studies were included in the meta-analyses if they were case-control or cohort studies which reported the association between maternal BMI and risk of ASD in offspring. To be included, studies had to have available results as an odds ratio (OR) or a relative risk (RR) with corresponding 95% confidence interval (CI), or sufficient data to calculate them. Non-human studies, case reports, conference abstract, review and studies that with insufficient data were excluded.

### Data extraction and quality assessment

The following data were extracted from each study: name of the first author, publication year, study name and period, study location, characteristics of participants (sex and age), number of cases and participants, the RRs or ORs with corresponding 95% CIs, confounding factors that being adjusted for in the analysis. We also extracted dose-relationship data for maternal BMI and ASDs risk, including the number of cases and participants (or person-years) and RR (95% CI) for each category of BMI.

The Newcastle-Ottawa Scale (NOS)^36^, which has been widely used for quality assessment of observational studies, was used to assess the methodological quality of included studies. This scale awards a maximum of nine points to each study: selection of the study groups (maximum 4 points), comparability of the study populations (maximum 2 points) and ascertainment of the outcome of interest (maximum 3 points). Studies that scored 0–3, 4–6, and 7–9 were considered as low, moderate, and high quality, respectively.

### Data analysis

To take into account heterogeneity between studies, a random-effects model was used to calculate summary RRs and 95%CI for non-reference (obese, overweight and underweight) versus the reference (normal weight) categories of BMI and for the dose-response analysis. The odds ratios (ORs) were considered equivalent to RRs considering that ASDs were sufficiently rare. Heterogeneity between studies was assessed by using Q statistics (significance level of *P* 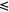 0.10) and *I*^*2*^ statistics^37^,^38^. *I*^*2*^ values of 25%, 50%, and 75%, was considered to be low, moderate, and high degrees of heterogeneity, respectively^38^. Subgroup analyses were conducted across a number of key study characteristics, including study location (Europe, USA), study design (prospective or retrospective), time of BMI measurement (pre-pregnancy, pregnancy), ascertainment of ASD (parental report, standard assessment), adjustment for maternal age (yes, no), child sex (yes, no), and birth year. We also performed sensitivity analysis to explore the effect of each individual study on the pooled result. Potential publication bias was examined by the Begg’s test^39^ and Egger’s test^40^.

For dose-response analysis, the method proposed by Greenland and Longnecker^41^ and Orsini *et al*.^42^ was used to compute study-specific slopes (linear trends) and 95% CIs from the natural logs of the RRs and CIs across categories of BMI. This method requires that the numbers of ASDs and persons/person-years for at least three BMI categories, and means or medians of the categories had to be available. When the median or mean BMI per category was not reported, the midpoint of the upper and lower boundaries was considered the BMI of each category. If the lower or upper boundaries for the lowest and highest category were not available, we assumed the length of these categories to be the same as the closest category. We used centered dose levels (each original non-reference dose minus the reference dose within a study) to summarize dose-response relation^43^.

A 2-stage random-effects dose-response meta-analysis was used to examine the potential trend between maternal BMI and risk of ASD^42^,^44^. We first applied restricted cubic splines with three knots in settled percentiles (10%, 50%, and 90%) of the distribution to model the possible association^44^. Then we derived the overall estimates with random effects by pooling the study-specific coefficient estimates and variance/covariance matrices that had been obtained in the first stage^45^. We calculated a *P*-value for nonlinearity by testing the hypothesis that the coefficient of the second spline was different from zero. For the study included in the dose-response analysis, we estimated a RR and corresponding 95% CIs for a 5 kg/m^2^ increase from 18 to 38 kg/m^2^ in BMI. STATA version 12.0 (StataCorp LP, College Station, TX) was used to analyze the data, and the significance level was set as 0.05 where otherwise specified.

## Additional Information

**How to cite this article**: Wang, Y. *et al*. Maternal Body Mass Index and Risk of Autism Spectrum Disorders in Offspring: A Meta-analysis. *Sci. Rep.* **6**, 34248; doi: 10.1038/srep34248 (2016).

## Supplementary Material

Supplementary Information

## Figures and Tables

**Table 1:** Characteristics of included studies of maternal BMI and ASD risk in the meta-analysis.

Author	Location	Study	Design	Age (y)	Time and ascertainment of BMI	Ascertainment of ASDs	ASD cases/Total participants	Confounding
Lyall 2011^17^	USA	The Nurses’Health Study II (NHS II)	Prospective cohort	NA	Pre-pregnancy Self-reported	Parental report	221/8, 498	Maternal age at baseline, race, income, body shape at age 20, cycle length, and age at menarche
Krakowiak 2012^16^	USA	Childhood Autism Risks from Genetics and the Environment Study (CHARGE)	Population-based case-control	2–5	Pre-pregnancy Extracted from medical record or self-reported	Identified through regional centers, providers/clinics, self referrals, and general public outreach. Validated with ADI-R and ADOS by trained clinicians	517/1, 004	Maternal age at delivery, race/ethnicity, education level, delivery payer, calendar time; child’s age at enrollment and gender, and catchment area
Surén 2014^18^	Norway	Norwegian Mother and Child Cohort Study	Prospective cohort	4–13.1	Pre-pregnancy Self-reported	Identified by questionnaire screening of mothers, professional and parental referrals of ASD suspection, linkage to the Norwegian Patient Register. Validated with ADI-R and ADOS	419/92,909	Parental education levels, child’s year of birth, and maternal parity
Moss 2014^9^	USA	The Early Childhood Longitudinal Study–Birth Cohort (ECLS-B)	Prospective cohort	4–5	Pre-pregnancy Self-reported	Parental report	100/4,800	Maternal age; child sex, birth weight, rates of height growth and weight gain; paternal BMI
Reynolds 2014^14^	USA	A prospective cohort study at a level-III neonatal intensive care unit	Prospective cohort	2	Pre-pregnancy Extracted from medical record	Identified by M-CHAT	14/62	Gestational age at birth, markers of sociodemographics
Gardner 2015^8^	Sweden	Stockholm Youth Cohort (SYC)	Prospective cohort	NA	At first antenatal visit Objectively measured	Ascertained by using ICD-9, ICD-10, and DSM-IV codes	6,420/333,057	Child’s sex, birth year, parity; parental age at the time of birth, maternal country of birth, paternal BMI, SES factors and parental history of psychiatric treatment
Xiang 2015^7^	USA	Kaiser Permanente Southern California (KPSC) Study	Retrospective cohort	1.5–2	Pre-pregnancy Extracted from medical record	Identified by M-CHAT, ascerntained by pediatric developmental specialist evaluations	712/68,837	Birth year

^*^ADI-R: Autism Diagnostic Interview Revised ADOS: Autism Diagnostic Observation Schedule M-CHAT: The Modified Checklist for Autism in Toddlers.

**Table 2:** Summary results of association between maternal BMI and ASD risk.

Variables	Underweight			Overweight			Obesity		
	No. of studies	RR (95%CI)	*I*^*2*^% (*P*-value)	No. of studies	RR (95%CI)	*I*^*2*^% (*P*-value)	No. of studies	RR (95%CI)	*I*^*2*^% (*P*-value)
Total	5	1.07 (0.93, 1.23)	0.0 (0.929)	5	1.28 (1.19, 1.36)	0.0 (0.521)	7	1.36 (1.03, 1.78)	77.5 ( < 0.001)
Location									
USA	3	1.07 (0.79, 1.46)	0.0 (1.000)	3	1.11 (0.93, 1.33)	0.0 (0.818)	5	1.27 (1.04, 1.57)	14.1 (0.324)
North Europe	2	1.07 (0.92, 1.25)	0.0 (0.352)	2	1.31 (1.21, 1.41)	0.0 (0.786)	2	1.50 (0.85, 2.63)	87.5 (0.005)
Study design									
Prospective	4	1.07 (0.93, 1.24)	0.0 (0.833)	4	1.30 (1.21, 1.40)	0.0 (0.785)	5	1.30 (0.88 1.94)	74.7 (0.003)
Retrospective*	1	1.07 (0.62, 1.84)	NA	1	1.11 (0.91, 1.35)	NA	2	1.35 (1.01, 1.78)	42.4 (0.188)
BMI measurement									
Pre-pregnancy	4	1.14 (0.87, 1.49)	0.0 (0.890)	4	1.15 (1.00, 1.34)	0.0 (0.807)	6	1.24 (1.06, 1.45)	2.7 (0.399)
First trimester	1	1.05 (0.90, 1.23)	NA	1	1.31 (1.21, 1.42)	NA	1	1.94 (1.72, 2.17)	NA
ASD ascertainment									
Parental report	2	1.07 (0.74, 1.56)	0.0 (1.000)	2	1.12 (0.77, 1.64)	0.0 (0.528)	2	0.92 (0.56, 1.52)	0.0 (0.421)
Standard assessment	3	1.07 (0.92, 1.24)	0.0 (0.648)	3	1.27 (1.16, 1.39)	15.6 (0.306)	5	1.48 (1.12, 1.98)	80.7 ( < 0.001)
Adjustment factors									
Maternal age									
Yes	3	1.05 (0.91, 1.22)	0.0 (0.996)	3	1.30 (1.21, 1.40)	0.0 (0.602)	5	1.51 (1.11, 2.07)	55.9 (0.059)
No	2	1.21 (0.82, 1.80)	0.0 (0.514)	2	1.16 (0.99, 1.36)	0.0 (0.458)	2	1.19 (1.00, 1.42)	0.0 (0.609)
Child sex									
Yes	2	1.05 (0.90, 1.23)	0.0 (0.989)	2	1.31 (1.21, 1.41)	0.0 (0.361)	3	1.60 (1.08, 2.36)	63.2 (0.066)
No	3	1.14 (0.87, 1.49)	0.0 (0.731)	3	1.16 (1.00, 1.35)	0.0 (0.755)	4	1.20 (1.02, 1.42)	0.0 (0.714)
Birth year									
Yes	3	1.07 (0.92, 1.24)	0.0 (0.648)	3	1.27 (1.16, 1.39)	15.6 (0.306)	3	1.41 (0.95, 2.07)	90.3 ( < 0.001)
No	2	1.07 (0.74, 1.56)	0.0 (1.000)	2	1.12 (0.77, 1.64)	0.0 (0.528)	4	1.31 (0.90, 1.90)	30.0 (0.232)

NA: not available Retrospective studies include case-control study and retrospective cohort study.
