# Supplementary material for: Maternal Body Mass Index and Risk of Autism Spectrum Disorders in Offspring: A Meta-analysis
Source: Sci Rep. 2016 Sep 30;6:34248. doi: 10.1038/srep34248 (PMC5043237; doi:10.1038/srep34248)
Supplement: Supplementary Information [file srep34248-s1.pdf]

Maternal Body Mass Index and Risk of Autism Spectrum Disorders in Offspring: A Meta-analysis

Author: Ying Wang, Shiming Tang, Shunsheng Xu, Shenhong Weng, Zhongchun Liu*

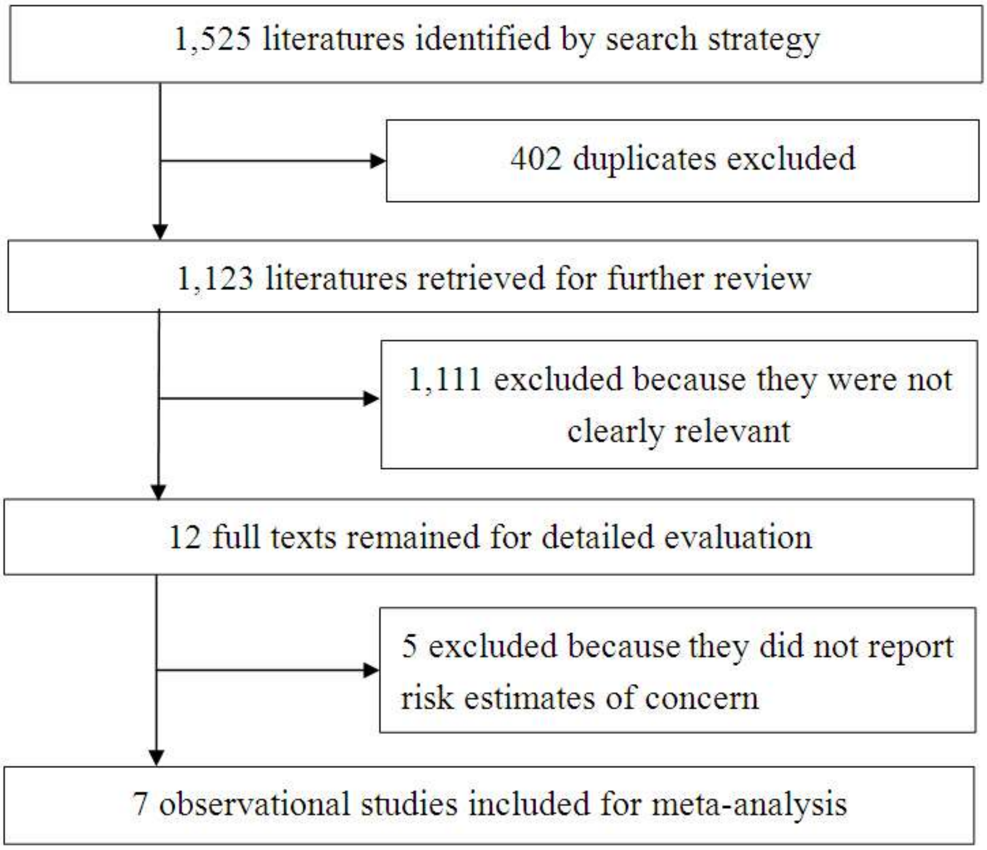

Supplemental figure 1. Flow diagram of the study selection process

Supplemental table 1. Methodologic quality of cohort studies included in the meta-analysis

Study	Representativeness of the exposed cohort	Selection of the unexposed cohort	Ascertainment of exposure	Outcome of interest not present at start of study	Control for important factor or additional factor[1]	Outcome assessment	Follow-up long enough for outcomes to occur	Adequacy of follow-up of cohorts	Total quality scores
Lyall 2011 [28]	1	1	1	1	2	0	1	1	8
Moss 2014 [9]	1	1	0	1	2	0	1	1	7
Surén 2014 [29]	1	1	1	1	1	1	1	1	8
Reynolds 2014 [25]	1	1	1	1	2	1	1	1	9
Gardner 2015 [8]	1	1	1	1	2	1	1	1	9
Xiang 2015 [7]	1	1	1	1	1	1	1	1	8
Study	Is the case definition adequate	Representativeness of the cases	Selection of Controls	Definition of Controls	Comparability of cases and controls[2]	Ascertainment of exposure	Same method of ascertainment for cases and controls	Non-Response rate	Total quality scores
Krakowiak 2012 [27]	1	1	1	1	2	1	0	1	8

[1,2] A maximum of 2 points were assigned to this item.
